# Priority measures for publicly reporting primary care performance: Results of public engagement through deliberative dialogues in 3 Canadian provinces

**DOI:** 10.1111/hex.13100

**Published:** 2020-08-03

**Authors:** Morgan Slater, Julia Abelson, Sabrina T. Wong, Julia M. Langton, Fred Burge, William Hogg, Matthew Hogel, Ruth Martin‐Misener, Sharon Johnston

**Affiliations:** ^1^ Department of Family Medicine Queen’s University Kingston ON Canada; ^2^ Department of Clinical Epidemiology & Biostatistics Centre for Health Economics and Policy Analysis McMaster University Hamilton ON Canada; ^3^ Centre for Health Services and Policy Research University of British Columbia Vancouver BC Canada; ^4^ School of Nursing University of British Columbia Vancouver BC Canada; ^5^ Department of Family Medicine Dalhousie University Halifax NS Canada; ^6^ Department of Family Medicine University of Ottawa Ottawa ON Canada; ^7^ CT Lamont Primary Health Care Research Centre ÉlisabethBruyère Research Institute Ottawa ON Canada; ^8^ School of Nursing Dalhousie University Halifax NS Canada

**Keywords:** patient‐oriented research, performance measurement, performance reporting, primary care, public engagement

## Abstract

**Objective:**

While public reporting of hospital‐based performance measurement is commonplace, it has lagged in the primary care sector, especially in Canada. Despite the increasing recognition of patients as active partners in the health‐care system, little is known about what information about primary care performance is relevant to the Canadian public. We explored patient perspectives and priorities for the public reporting of primary care performance measures.

**Methods:**

We conducted six deliberative dialogue sessions across three Canadian provinces (British Columbia, Ontario, Nova Scotia). Participants were asked to rank and discuss the importance of collecting and reporting on specific dimensions and indicators of primary care performance. We conducted a thematic analysis of the data.

**Results:**

Fifty‐six patients participated in the dialogue sessions. Measures of access to primary care providers, communication with providers and continuity of information across all providers involved in a patient's care were identified as the highest priority indicators of primary care performance from a patient perspective. Several common measures of quality of care, such as rates of cancer screening, were viewed as too patient dependent to be used to evaluate the health system or primary care provider's performance.

**Conclusions:**

Our findings suggest that public reporting aimed at patient audiences should focus on a nuanced measure of access, incorporation of context reported alongside measurement that is for public audiences, clear reporting on provider communication and a measure of information continuity. Participants highlighted the importance the public places on their providers staying up to date with advances in care.

## 1 INTRODUCTION

Performance measurement is commonplace in health‐care systems worldwide, used for quality improvement efforts,^1^, ^2^ public accountability,^3^, ^4^, ^5^, ^6^, ^7^ patient engagement,^8^, ^9^, ^10^ research and informed decision making.^11^ Public reporting of performance measures is fundamental to achieving several of these goals. While public reporting on hospital‐based procedures and care has been growing over the past two decades, public reporting of primary care performance measures has lagged behind; however, countries such as the United Kingdom, United States and Australia have led efforts in public reporting of primary care performance.^12^, ^13^, ^14^ Canada, like many countries, has experienced two decades of primary care reforms in health service delivery.^15^, ^16^, ^17^, ^18^ While the majority of Canadians receive most of their health care from primary care providers, there is a paucity of publicly reported information to engage members of the public,^19^ and no federal organizations are mandated to publicly report on primary care performance. While the Canadian Institute for Health Information has an active health system performance public reporting site,^20^ it includes very few measures of primary care performance. Provinces, who are responsible for the delivery of primary care services, also do not consistently publicly report performance in primary care.^19^

Patient engagement at the level of care decision making has been shown to improve both patient‐ and health system‐level outcomes.^21^, ^22^ Canada's Strategy for Patient‐Oriented Research (SPOR) highlights the importance of incorporating patients as active partners across the continuum of research to health system transformation^23^ and primary care has embraced patient engagement in the realm of quality improvement.^24^, ^25^, ^26^ While public perception of how the health system is performing is influenced by context and culture,^27^, ^28^ patients and citizens have reported that public reporting of primary care performance in Canada could support community advocacy and health system decision making and increase the public's trust in the care they receive.^29^ While easily accessible data that engage the public in performance reporting is deemed important,^12^, ^13^, ^30^ however, little is known about the Canadian public's perspective on which specific information related to primary care should be shared with them.^31^, ^32^, ^33^

The objective of this manuscript is to examine patient perspectives and priorities for the public reporting of primary care performance measures in the Canadian context. This project is part of a larger programme of research to improve the science and reporting of primary care performance in Canada^29^, ^34^ and builds on our previous analysis that described how the public might use reports on primary care performance.^29^

## 2 MATERIALS AND METHODS

We conducted deliberative dialogues, a well‐established approach for engaging the public in complex issues,^35^, ^36^, ^37^ in three provinces that were selected for their varied approaches to primary care reform: British Columbia (BC), Ontario (ON) and Nova Scotia (NS). The methods for these dialogues have been previously reported.^29^ Briefly, six day‐long deliberative dialogue sessions were held between January and May 2016. Two sessions were held in each of three distinct regions: (a) Fraser East, British Columbia, (b) Eastern Ontario Health Unit, Ontario and (c) Central Zone, Nova Scotia. The regions were selected for their similarities in socio‐demographics (eg, ethnicity, age and socioeconomic status)^38^ and their differences in health reform, the availability of primary care physicians and proportion of the population with a primary care provider. One session in each region was conducted with patients with complex needs (multiple comorbid conditions) while the second event was for those with two or fewer medical conditions. The study was approved by the research ethics boards of the University of British Columbia, Nova Scotia Health Authority, Ottawa Hospital and Bruyère Continuing Care (Ottawa).

### 2.1 Participants

We recruited patients 18 years of age or older, most of whom participated in a waiting room survey of patient experience^39^ at their primary care practice and consented to being contacted for research opportunities. We used the patient experience survey to obtain information on respondents' age and medical conditions, and from this convenience sample, we purposefully recruited participants who spoke English with diverse ages, gender, types of chronic conditions and the practice where the individual received care. We used the number of chronic conditions as a proxy measure of medical complexity to recruit participants with diversity in experience with the health system. Participants received a $75 honorarium for their time, received meals during the event and reimbursement for transportation.

### 2.2 Structure of session

Each deliberation session was conducted over the course of a single day in a central location within each study region. Dialogue sessions were jointly facilitated by research team members with expertise in primary care performance measurement, patient experience, public performance reporting and deliberative dialogue methodology. Prior to each session, participants received an information package which contained the project objectives, background information about primary care performance measurement, including the rationale for public reporting, definitions of key terms, and examples of the most commonly used performance domains and examples of different indicators within that domain (Table 1). Indicators were selected among those publicly reported in the past decade or ones from our larger study to populate dimensions not usually publicly reported. We ensured indicators represented diverse data sources including patient reported measures and those routinely collected in administrative data.

**TABLE 1:** Examples of the most commonly used performance domains and indicators provided to participants as background information

*Access*: The ease with which clients or patients can initiate contact with their primary health‐care provider for a new or existing health problem	
How it is measured	Percentage of respondents who report having a family physician or nurse practitioner that they see for their regular care, or when they are sick
	Percentage of patients who report that they were able to see their family physician or nurse practitioner on the same or next day
	Percentage of patients who report that getting medical care in the evening, on a weekend or on a public holiday was difficult
	Percentage of patients who report that, when they call their regular family physician's office with a medical question or concern during regular office hours, they get an answer on the same day
*Patient‐Centred Care*: Providing care that is respectful of and responsive to individual patient preferences, needs and values, and ensuring that patient values guide all clinical decisions	
How it is measured	Percentage of adults with a regular family physician or nurse practitioner who said their regular health‐care provider always explains things in a way that is easy to understand
	Percentage of patients who report that their family physician, nurse practitioner or someone else in their medical office spends enough time with them
	Percentage of patients who report their family physician, nurse practitioner or someone else in the medical office involves them as much as they want in decisions about their care or treatment
*Continuity*: The delivery of services by different providers in a timely and complementary manner such that care is connected and coherent within an acknowledged care plan	
How it is measured	Percentage of total primary care visits that are made to the patient's primary family physician or nurse practitioner
	Percentage of patients who report that there were often times when the health‐care provider they were seeing did not have access to their recent tests or examination results
*Effectiveness*: Providing care that works and is based on the best available scientific information	
How it is measured	Percentage of eligible patients aged 50 to 74 who had a faecal occult blood test (FOBT) within the past two years, sigmoidoscopy or barium enema within five years or a colonoscopy within the past 10 years
	Percentage of people with diabetes who had a serious complication from it in last year
	Percentage of people with high blood pressure who had a BP check recorded in the last year

The agenda for each session was structured around three distinct topics: prioritizing primary care performance dimensions and indicators for public reporting; uses of performance information; and effective reporting formats. We opened each session by asking participants what they would tell someone moving to their region to help them understand the quality of the primary care they might find there. The first hour of the session was then spent reviewing background material and orienting the participants to primary care and performance measurement. Throughout the session, we presented scenarios, illustrative examples and international comparisons including reporting in Australia, the UK and Canada. Interactive case discussions were used to both familiarize the participants with the concepts and allow them to share their experiences and opinions.

The focus of this analysis is the discussion surrounding the prioritization of primary care performance dimensions and indicators for public reporting. Participants were provided with worksheets listing and defining key performance dimensions for primary care (access, patient‐centred care, continuity, comprehensiveness, technical quality of care, safety, service integration and health equity, Table 2), based on an existing framework for comprehensive performance management in primary care.^40^ This first discussion introduced participants to this broad and comprehensive view of primary care performance, with examples of indicators for each of these dimensions to help make the concepts more concrete. Participants were then asked to rank the importance of collecting and reporting on these primary care performance dimensions (ranging from not at all important to very important) followed by a group discussion of their ratings and corresponding rationales.

**TABLE 2:** Dimensions and indicators of primary care performance with ranking scale used by participants

Ranking	Not at all important	Slightly important	Important	Fairly important	Very important	No opinion
Dimension						
Indicator						
Access						
*Patients have a regular family physician or nurse practitioner that they see for check‐ups or when they are sick*						
*Patients can see their family physician or nurse practitioner on the same or next day when they call for an appointment*						
*Patients can get medical care in the evening, on a weekend, or on a public holiday through their family physician or nurse practitioner*						
*Patients can call their regular family physician's office with a medical question or concern during regular office hours and get an answer on the same day*						
Patient‐centred care						
*Patients think their family physician or nurse practitioner always explains things in a way that is easy to understand*						
*Patients think that their family physician or nurse practitioner spends enough time with them*						
*Patients think that their family physician or nurse practitioner involves them as much as they want in decisions about their care or treatment*						
*After seeing their family physician or nurse practitioner, patients feel more confident in dealing with their health problems than before their visit*						
Continuity						
Comprehensiveness						
Technical quality of care						
*Patients are offered screening for various health problems according to recommended practice*						
*Family physicians, nurse practitioners or an appropriate person in the office discusses the impact of health and non‐healthy foods on patients’ health*						
*Patients with diabetes have their blood sugar at the recommended target level*						
*Patients with high blood pressure had a blood pressure check in the last year*						
Safety						
Service integration						
Health equity						

Next, participants further explored three dimensions of primary care performance and related indicators purposefully selected in advance of the meeting: *access*, to capture an area perceived as highest priority for public reporting and a focus of reform efforts over the previous decade across the country^15^;*patient‐centred care*, selected as a key pillar of the next wave of primary care reforms towards medical homes that is not routinely public reported and an important component of patient experience in primary care^41^, ^42^; and *technical quality of care*, a dimension aimed to show prevention and treatment activities. Participants were asked to rank on a worksheet the importance of each dimension's specific indicators for public reporting (Table 2) followed by a group discussion of the shared rankings.

### 2.3 Analysis

Each deliberation session was recorded and transcribed. The transcripts were read using immersion crystallization by two team members experienced in qualitative analysis (MH, SJ), one who was present for all sessions and was responsible for data cleaning.^43^ A coding template, informed by the study objectives, our project framework for comprehensive performance measurement for primary care,^40^ and initial review of the transcripts, was inductively developed by the interdisciplinary analysis team, which included the four team members who designed and facilitated the deliberation sessions with backgrounds in nursing, medicine, epidemiology and health policy. The coding scheme was then applied to the transcripts by the same two team members (MH, SJ). Two researchers (MS, SJ) independently performed a thematic analysis on the content reports for each code to identify recurring themes (defined as those arising in three or more dialogue sessions). Using Lincoln and Guba's trustworthiness framework,^44^ we examined *confirmability* of the statements at frequent check‐ins during the deliberative dialogues. We also ensured *credibility* of the analysis by having additional team members provide substantive feedback on the themes and interpretations, and we examined *authenticity* by examining the range of participants' realities by seeking out disconfirming statements and unexpected findings. Our analysis was informed by a framework for comprehensive performance measurement for primary care^40^ which had informed the study design and presentation to participants. The researchers initially analysed four code content reports independently and found near perfect agreement in identification of themes. They then completed the analysis of the remaining reports. Shared themes were also mapped to identify if there were any specific to patient groups with more medically complex (defined as having two or more medical conditions) or less complex conditions.

## 3 RESULTS

Fifty‐six participants were involved across six deliberative dialogue sessions. Participants were mainly Caucasian, between 20 and 81 years old, with a range of medical conditions, from none to 10 different conditions (Table 3).^29^ Gender was balanced across participants in the dialogues except for those in Nova Scotia where the participants were predominantly female. No recurring themes were limited to or significantly more prominent within the patient groups with more or less complex conditions. Participants provided their perspectives about both the importance of specific dimensions of performance and indicators as well as the value of reporting various measures to the public.

**TABLE 3:** Demographic characteristics of dialogue participants^29^

	Less medically complex patients			Medically complex patients		
	BC (n = 6)	ON (n = 11)	NS (n = 11)	BC (n = 14)	ON (n = 10)	NS (n = 6)
Number of female participants	2	8	10	7	5	5
Age, years, mean, (SD)	n/a^a^	n/a^a^	n/a^a^	64.1 (11.9)	61.4 (10.9)	56.7 (12.5)
Number of chronic conditions, mean (SD)	1.3 (0.5)	0.7 (1.1)	0.3 (0.5)	4.9 (2.1)	5.4 (2.4)	4.7 (2.5)

^a^ Data unavailable for 13 of the less medically complex participants who were recruited through an online volunteer ad.

### 3.1 Key dimensions of primary care performance

We present themes arising for each primary care performance dimension and specific indicator in the order in which the dimensions were presented to dialogue participants. There was little discussion among participants surrounding the dimensions of comprehensiveness, safety, service integration, or health equity and as such are not discussed.

### 3.2 Access

Many participants noted that without access to primary care, other indicators of performance are less relevant: *If you can't get access, how do you evaluate the other priorities?* [ON_2]. Across all dialogue sessions, access to primary care was clearly seen as the most important indicator to publicly report, a foundational marker for how the health system is working.

When presented with specific indicators to measure access, having access to a family physician or primary care provider was clearly important:

*I believe that everybody should have a general practitioner or family doctor and I think that there needs to be some way to track that. … family doctors control the whole thing …* [BC_2]

However, there was debate about the value of a same‐day appointment as a meaningful measure of performance. Most participants suggested that same‐day access was context‐specific, depending on the nature of the problem.

*I mean, if you have a cold, it’s going to go away on its own. Some of the things that you have wrong with you people can wait to see a doctor*. [ON_2]

Participants felt that a same‐day response to phone calls was too short of a time frame. Context again was important: an acceptable time frame for a response varied from the same day for urgent matters, to anywhere from a week to a month for less urgent matters. Participants also recognized that these needs could be met using other parts of the health‐care system:

*You’re going to call your busy doctor’s office when he’s with patients [and not expect him to] call back the same day necessarily because there are other telephone groups for that. There’s the Emerg Department if it’s really important. There’s the nurse line. There’s things, resources that can be used to get an answer to a question rather than interrupt your doctor’s busy day*. [NS_2]

After‐hours access was revealed to be a priority to measure for most individuals in each group:

*I just wanted to say that Saturdays and Sundays shouldn’t be an issue. Today people are working—my daughter works in mental health, her weekends are Mondays and Tuesdays. Doctors would be the same thing… a week is a week, so if you’re sick on a Saturday or a Sunday, you should have access to healthcare. So, for me, Monday or Saturday there’s no difference*. [ON_2]

However, there was debate around where this after‐hours care should be provided. Very few participants suggested that they needed to see their own physician: *I mean, I believe we should have access to medical care, but I don't believe that our primary physician should be available 24 hours a day, 7 days a week* [ON_2], but felt it was important to have after‐hours options within their own primary care practice:

*So if I see my physician when I book an appointment for my physical or whatever ahead of time, and she’s quite accessible in that respect, but [if] I was to call and say, ‘I need to see somebody tomorrow’ if she doesn’t have any availability, they’ll hook me up with somebody else who works in the clinic, they have access to all my information*. [ON_1]

On the other hand, many participants felt that regional access to emergency or after‐hours care was sufficient and important to measure and report on: *I think information [that] is important is does that area have a walk‐in clinic that you can go to instead of the ER Also, if there [is] an ER there, cause I don't think that anybody would expect that they could contact their own GP anytime of the day or the week*. [BC_2].

In dialogues within each province, participant perceptions of access were shaped by the view that primary care is a scarce, valuable resource, with limits on what it could and should accomplish. There was a clear sense of participants seeing and valuing family physicians as people with their own needs: *Physicians are also human beings, right? They have to enjoy their evenings and if it's on the weekend, we can go to the walk‐in clinics, anyway* [NS_2].

### 3.3 Patient‐centred care

Across all groups, patient‐centred care was also viewed as an important dimension to publicly report:

*For me it does represent a measure that the other things are happening in a positive sense. So, I keep coming back to collecting and reporting and would I want to read about this if I was trying to judge whether there was good healthcare system in an area. Yeah, I would use it as a measure, as an overall measure* [NS_2]

However, when presented with four potential indicators of patient‐centred care (Table 1), participants in each group felt that enough time was an important measure of the patient experience. Enough time allows them to explain what their issue is and to understand what the clinician is telling them.

*We hear stories where patients go in and they say, ‘I was in there no more than five minutes. I don’t think he’s listened to me’ or something like that. Maybe make them aware, make the system aware that we might have to spend more time with each patient. …I think it has to be measured. It’s very important for me because I think—I want to make sure that my doctor, my physician listens to me, takes the time to explain it back to me and any other questions …*. [ON_2]

The concept of enough time was also interpreted by participants to mean the primary care provider was listening enough to find out what the patients’ issue/health concern was.

*One of the really important ones there would be do I feel a doctor took enough time with me because odds are, if I feel a doctor took enough time with me, I think that would indicate that I had the time to understand the discussion about what my treatment and being part of that decision might encompass a few more right into that list*. [BC_2]

*If you don’t have enough time to sort of talk about whatever your issue is, they might not actually find what the real problem is*. [NS_2]

However, in some dialogues there was disagreement about patients perceiving enough time with their provider as a priority performance measure and there was debate about which other indicator(s) should be reported in all the groups:

*I put the first and the last one as very important [explains things well and feels more confident] because if we concentrate on those two questions, they’re actually almost including those other two things [enough time and involved enough] and I think that if you understand what your physician is talking to you about and then afterwards you have a level of confidence that you feel involved in your own health, I think those are the important things to feel confidence about as person when you’re making your decisions*. [ON_2]

Having clinicians ‘spend enough time with them’ was also seen to be subjective as patients’ preferences for ‘enough time’ could vary from person to person:

*…. because you could spend two hours explaining a very complicated thing to a person with big words, or you could spend ten minutes explaining very complicated things in simple terms that anybody can understand, and I think—and also I think that enough times it’s very subjective to whoever’s rating it*. [ON_1]

Overall, these quotes suggest that communication and interaction between patients and their providers is important for patients to understand and have confidence beyond the primary care visit.

### 3.4 Continuity

A recurrent theme across groups was that continuity of information is an important system‐level measure that significantly impacts primary care performance:

*…because we’re looking at many different specialists, chiropractors, dentists—all of those people should have the same [health] information [about me] and it doesn’t happen*. [BC_1]

Interestingly, a system that enabled continuity of information was seen as a way to become better at meeting patients’ health‐care needs. Past medical history is information that could be relevant to numerous providers treating the same patient:

*I think continuity and patient‐centred care are tremendously important particularly as we get more and more technical within the delivery of health care that the patient can actually get lost, they become an average, you know, rather than [a person] coming in whose had several health problems that are related, and should be known about when we’re treating him with this cough… as we get more and more technical, we have to keep focusing on the patient and that information gets passed from the general practitioner to the physiotherapist, so we’re actually becoming more efficient in looking at this person not having to cover it again to find out that they had a broken leg, like, three years ago*. [NS_2]

Participants did not bring up the need for increased privacy of the medical information. Rather, they suggest health‐care providers should know more about their care in different parts of the system: *So, I think that's how your…that's reporting back to the public about that. I mean, first of all, it has to be in place, that there is a place where all information for a particular patient goes and I can ask this as an individual because I’m the patient, but then all of my caregivers can access it as well and I realize that there's all sorts of confidentiality issues in there, but I think that the service is more important than the confidentiality*. [BC_1].

### 3.5 Technical quality of care

When discussing measuring and reporting on the delivery of primary care services, a recurrent theme across groups was the lower value placed on publicly reporting performance measures for aspects of care that are seen as being outside of the care of primary physicians or that could be delivered in other parts of the system. These measures included whether the family physician had counselled on healthy diet or medication side effects. As one participant stated: *I just think that our doctors who don't take huge courses in nutrition, but there are young people who go there and that's all they study and then become really good at their trade and … we should all have access to them and we could allow the doctors to be doctors. I think we're expecting doctors to do everything. …Then I just think there's a way to teach dietetics and nutrition with the community health*. [NS_1].

Concern was expressed in all groups that some of these measures, including ones which would come from information in patient health records or administrative data, may be less valuable as a measure of primary care performance because they are ‘patient‐dependent’. Patients understand that these metrics may not reflect performance of the primary care system or providers because patients can choose not to follow recommendations or seek care:

*…a lot of the screening practices are currently being reported publicly … and I think that here what’s important is, is an indication that your family practitioner is up‐to‐date and following the recommendations. Not that your patients necessarily have to follow them all. They may say, ‘No thank you’. Lots of people don’t want to have a colonoscopy and all that,* [ON_1]

*You cannot take someone to the doctor and get them to check their blood. Some people just don’t like to go to the doctor*. [NS_2]

Not only may these measures reflect patient choice, some participants felt that these measures could be considered unfair as a rating of clinician performance as they might also be affected by poor self‐management: *My bottom line is, proactive is the way to go. Some of the people don't do that, that's the problem, then they blame the doctor. You can't blame the doctor. You've got to be proactive*. [BC_1] This suggests patients are aware that performance metrics may not only be about one clinician's abilities but a complex host of factors including, for example, the patient's willingness and ability to obtain the recommended tests.

Interestingly, in dialogues in Ontario and Nova Scotia, participants raised the importance of continuing professional development as an important dimension of quality care: *I do think that's important because things are changing. New research is being done on different diseases and stuff like that. So how up‐to‐date are—like I know my particular doctor, we go in with a question, she'll say to us, “Well, I don't know anything about that, but I will check into it.” And within a week, she's calling us back, “Look I found this out. And you may want to check into this.” I’m fortunate with the doctor I have that she will check into those things. But how often are these doctors going in for professional refresher courses, or going back for a month or two to school to see what these new practices are, or a new way of doing things is my concern*. [NS_1] This topic was unprompted by facilitators and came up in three separate dialogues.

## 4 DISCUSSION

Despite the proliferation of publicly reported information on health system performance, there are few studies that focus on understanding the patient point of view.^31^, ^32^, ^33^ In this study, we found that patients identified clear priorities for primary care performance measurement and reporting that could be taken into account when deploying information on health‐care performance. Specifically, measures of access to a family physician, patients perceiving that their primary care provider spent enough time with them, and continuity of information across all providers were identified as high priority indicators of the performance of the primary care system. Interestingly, several other commonly used performance measures, such as rates of cancer screening or regular blood pressure measurements, were viewed as being patient‐dependent and as such were not felt to be an accurate measure to evaluate the health system or the primary care provider's performance. Participants were concerned that primary care physicians were up to date with current research evidence and highlighted the importance of continuing professional development. Our findings were relatively consistent across the three provinces included in our deliberative dialogues, despite the diversity in primary care policies and regional health concerns.^38^

Access to primary care was found to be fundamental to understanding the performance of the health‐care system from the patient's perspective. This is not surprising and may be a function of the common role of primary care as the entry point for most patients into the health‐care system across the country. As such, this finding might not be replicated in health‐care systems in which a primary care provider does not function as an access point and gatekeeper for the whole system. While all participants had a regular family physician and most were recruited during visits to their own primary care practice, this finding may also have been shaped by the commonly held perspective that it is difficult to get a family physician or nurse practitioner for some people or in some regions in Canada,^29^ where 83%, 90% and 89% of the population in BC, ON and NS, respectively, report having a regular source for primary care.^45^

Participants provided insight on the value of specific measures of access: optimal access might depend on the context of the health issue rather than a single measure of same‐day access. This may reflect people's experience in primary care involving care across a wide range of concerns or the longitudinal relationship‐based nature of primary care in which patients typically are cared for by one person who they may come to see as having needs of their own. It might also reflect the view which emerged in multiple dialogues about primary care as a publicly funded scarce resource rather than a consumer‐oriented service. While there was support for the importance of after‐hours access to care, the debate about its value as a performance measure for primary care highlighted similar tensions recognising a need for some form of available primary care while balancing the view of it being a limited resource and respecting primary care providers as individuals with limits as well.

The emphasis on measuring and reporting on continuity of information rather than on continuity of care with a single provider was notable. Patient understanding of continuity of care appears to go beyond the narrow definition often used in primary care; rather than care being concentrated in a single provider, it seems that patients see continuity as an expectation of shared information and care management.^46^ This may be related to an increased push to allow patient access to electronic medical records.^47^ Participants viewed a wide range of community‐based services as primary care providers and thus a measure that would capture availability of services in the community—not limited to those solely available through their primary care practice—might better capture people's experiences and expectations. Coordination and continuity of information both within and across primary care providers took precedence over the protection of privacy of health information. Participants did not raise concerns about privacy or confidentiality, rather they expected that providers should be able to access all information relevant to their care. Innovations to our health information systems lag due to privacy concerns, with privacy being prioritized over access to information within the circle of care.^48^, ^49^ However, patients clearly expect technology to connect those providers who are caring for them. Overall, this may point to a need for a publicly reported indicator on information continuity, which would reflect health system performance not just primary care.

The recurring theme of not valuing performance measures which were dependent on patient action, including typical ‘process measures’, such as taking blood pressure or rates of cancer screening, which are often seen as within the ‘control’ of primary care providers, suggests that, from the patient perspective, these may not be valuable measures of primary care performance given that both patients and providers have a shared responsibility in these activities. Similar concerns about provider control of performance measures have been raised by primary care providers.^50^ A recent multi‐stakeholder engagement to develop a practical yet comprehensive instrument to evaluate primary care performance from patients’ perspectives did not include many of the specific process measures traditionally used for this purpose.^41^ No core set of process measures emerged as a priority litmus test of performance for primary care reinforcing recent work suggesting that simply aggregating a range of disease‐specific performance measures does not provide an adequate measure of high‐quality primary care.^51^ Given that participants saw primary care providers as humans with their own needs, this may also speak to patients having conflicted feelings about performance reporting, not wanting to ‘blame’ their physician. Patient understanding of these measures is likely focused around their own experiences with the health‐care system; patients view these measures as measures of their self‐care rather than an indicator of the system overall. If the goal of public reporting is to increase public engagement in improving the system, we will need to educate the public as to why these measures speak to the overall performance of the system and frame the use of these measures in the context of a learning health system^52^ rather than as punitive, given the understanding of patient agency in many of these measures. However, it is not clear that such explanation or qualification of measures is required given that not all information will be accessed by the general public; rather, effective public reporting of performance measurement needs to be simple and layered with different information targeted to reach different audiences (eg patients, health‐care providers, health system managers).^12^, ^13^, ^30^

There are a variety of reasons to report primary care performance to the public, including accountability for public funds, and public engagement in shaping the system.^1^, ^2^, ^3^, ^4^, ^5^, ^6^, ^7^, ^8^, ^9^, ^10^, ^11^, ^29^ If notions about individual responsibility for health and experiences seeking and receiving care shape people's perception of the value of specific measures, the measures best suited for these purposes are influenced by the system and culture in which they are being deployed.^53^, ^54^ Without an accompanying understanding of the context in which the measures occur and the role of social determinants in a person's health‐promoting behaviour, some measures seeming to reflect patients’ ‘choices’ may exacerbate social misconceptions. Patients recognize that they may not be the primary users of publicly reported performance data and see public reporting as a means to fuel accountability and drive improvement among providers.^29^ Assessing relative performance by different patient groups will allow providers to determine what additional strategies are needed to support successful outcomes among these patients. This also speaks to the importance of including information about the context of primary care practices in a province or region, changing the focus from single providers to a wholistic view of the system. The findings of this study are particularly relevant given the interconnectivity of the primary health‐care system with the greater health‐care system.^55^, ^56^, ^57^, ^58^ Strong primary care systems improve the overall health of the population.^58^

The public's interest in performance measures for primary care may be intertwined with notions of individual responsibility and agency which is perhaps a stronger influence in primary care reflecting the longitudinal, low urgency nature of primary care. The notion that individuals are independent agents in primary care with responsibility for their outcomes may have also been linked to the view of many of the measures as subjective and thus not relevant to one's own view of the performance of the primary care system. Supporting the view of primary care as enabling individual agency was the finding that measuring patient‐centred care was important and, more specifically, spending enough time with patients was prioritized. Rushing the patient‐clinician interaction might undermine that opportunity to be an individual with unique needs and preferences in primary care.

It was particularly noteworthy that participants raised the importance of continuing professional development (CPD) as an important dimension of quality without any prompting or background material that raised this issue. While we do not know what motivated this discussion, we can offer some possible explanations. The public may be aware of the breadth of knowledge required of primary care practitioners and the need to remain current with continually advancing to provide the best care for their patients. Trust in professional credentials has been called a type of social or institutional trust.^59^ It is seen as a functional trust that allows patients to get the minimum out of an encounter with their provider, in part due to the vulnerability of someone who needs help.^60^ Primary care usually involves longitudinal relationships and higher levels of trust in the motivations or intentions of the provider.^60^, ^61^ While patients may trust primary care providers’ intentions,^62^, ^63^ CPD credentials might support trust in the abilities of the provider they will see. This suggests further research on CPD activity and primary care performance may be warranted.

It is important to note limitations associated with this study. Each deliberative dialogue was held over one day and included time for both education and discussion of complex topics. As a result, the depth of insights and perspectives we were able to gather was limited. Given the significant time required for education about public reporting, we recommend that future studies in this area allow for more time for these discussions. In addition, while participants were asked to assign priorities to all dimensions of care, there was little discussion of some dimensions (ie comprehensiveness, safety, service integration or health equity). This lack of discussion should not be viewed as an indicator of their lack of importance. Further, participants did not have the same opportunities to discussion indicators for all dimensions as discussions were focused on three domains (access, patient‐centred care and technical quality of care). Participants were provided with specific indicators for certain dimensions that may have influenced the discussion. Additionally, the majority of participants in this study had a regular primary care provider and the views of patients who have more difficulty accessing primary care might be different. The participants in this study mainly discussed family physicians as primary care providers; while nurse practitioners (NPs) are playing an increasingly important role in primary care in Canada,^64^ there are few NPs practicing in these regions (especially in NS and BC),^18^ and thus, participants may not have had the opportunity to see an NP. Finally, it is important to note that the sample is not necessarily representative of the general public.

## 5 CONCLUSION

Several key measures of primary care performance emerged as priorities for public reporting across deliberative dialogues in three different regions across Canada. Performance reporting serves different functions for different audiences; to be relevant to the patient perspective, public reporting aimed at reaching patient populations could focus on access, with a more nuanced approach to timeliness, the presentation of context alongside measurement that is designed specifically for a public audience and clear reporting on communication with health‐care providers. Interestingly, participants highlighted the importance of continuing professional development to ensure that primary care physicians were up to date with current evidence. Our finding that continuity of information is important to patients goes beyond simply developing measures for reporting and is a call to action to set up the infrastructure to support this need. Overall, the selection of optimal performance measures for primary care may be significantly influenced by culture and health policy context.

## CONFLICTS OF INTEREST

None declared.

## AUTHORS' CONTRIBUTIONS

JA, SW, JL, FB, WH, RMM and SJ conceived and designed the study. MS, MH and SJ completed the data analysis. All authors contributed to the interpretation of the data. MS drafted the manuscript. All authors critically revised the manuscript, gave final approval and agree to be accountable for the work.

## Data Availability

The data that support the findings of this study are available from the corresponding author upon reasonable request.
